# Collagen microsphere based 3D culture system for human osteoarthritis chondrocytes (hOACs)

**DOI:** 10.1038/s41598-019-47946-3

**Published:** 2019-08-28

**Authors:** P. Yeung, K. H. Cheng, C. H. Yan, B. P. Chan

**Affiliations:** 1 Tissue Engineering Laboratory, Department of Mechanical Engineering, The University of Hong Kong, Pokfulam Road, Pokfulam, Hong Kong; 2 Department of Orthopaedics & Traumatology, Li ka Shing Faculty of Medicine, The University of Hong Kong, Pokfulam, Hong Kong

**Keywords:** Biomaterials - cells, Cell biology

## Abstract

The current study aims to evaluate collagen microencapsulation as an *in vitro* 3D culture platform for human osteoarthritic chondrocytes (hOACs), and to exemplify its feasibility in screening potential disease modifying factors. We first isolated and expanded hOACs from osteoarthritis (OA) cartilage samples harvested from multiple patients during total knee replacement (TKR) surgery. These cells were microencapsulated into collagen microspheres for subsequent 3D cultures. The change in chondrocyte phenotypes and OA phenotype was evaluated over time, using 2D monolayer culture and traditional 3D pellet culture as controls. The hOACs in the 3D collagen microsphere model resumed their *in vivo* phenotypes when compared to 2D monolayer. When compared with the 3D pellet model, the 3D hOAC-collagen microsphere model better recapitulated the OA phenotypes. We further demonstrated the responsiveness of the microencapsulated hOACs towards a number of external factors altering the chondrogenic phenotype, corroborating with previous studies. The hOAC encapsulated collagen microspheres better maintained the hOAC phenotype than the traditional 2D monolayer and 3D pellet cultures. The feasibility to use this hOAC-collagen microsphere *in vitro* model as a screening platform for disease-modifying agents has been demonstrated, contributing to future development of OA therapeutics.

## Introduction

### Osteoarthritis – chondrocyte phenotype-altering degenerative cartilage disease

Osteoarthritis (OA) is a degenerative joint disease that is very common among the elderly. The causes of OA are multifactorial and remain elusive. As cartilage lacks the intrinsic ability to repair itself, intervention would be required to slow down or stop disease progression. However, very limited efficacy could be benefited from symptom relieving measures such as physiotherapy and pain management drugs^1,2^. For terminal stage patients, joint replacement become the most cost-effective surgical interventions. However, the risk of complications in elderly is substantial^3^ and second surgery is probably required in most patients given the life span of an implant is only about 10–15 years^4^. Disease modifying treatments for osteoarthritis are therefore very essential in postponing the time for joint replacement as much as possible. Currently, there are some candidate drugs under development such as anti-degeneration agents including Matrix metalloproteinases (MMPs) and a disintegrin and metalloproteinase with thrombospondin motifs (ADAMTS) inhibitors, cartilage regeneration inducing agents such as sprifermin, anti-bone resorption agents and anti-inflammatories agents involving antibodies and small molecules. Yet the outcome of clinical trials for most of these candidates are so far disappointing and insignificant^5^, leading to wastage of tremendous amount of effort and resources. This suggests the urge need for improving our understanding towards the OA pathophysiology and selecting the most effective candidates for OA therapeutics. Establishing physiologically relevant pre-clinical models which enable precise and accurate study of disease phenotypes and allow high-throughput drug screening is therefore of utmost importance. Given that the heterogeneous nature of the disease, patient-specific models would be even more desirable as they could hopefully give more insights of this complex disease.

### *In vitro* models for osteoarthritis research

Existing *in vitro* models include monolayer culture, 3D scaffold culture and cartilage explant culture. Monolayer culture of chondrocytes is simple and is the most commonly used one but cells usually lose their *in vivo* phenotypes when they are isolated from the tissues^6^. Pellet culture is the most commonly used 3D culture model. However, due to the scarce cellularity in cartilage and limited proliferation power of chondrocytes, imposing a huge number of cells into a single pellet is neither practical nor economical for high throughput screening. The limited supply is also the major concern of explant culture approach which also has the issues of central necrosis. With the advance in cell biology and biomaterials, tissue engineering approaches are being brought to the forefront of *in vitro* model development. By using biocompatible materials as scaffold, tissue engineer could establish 3D culture with *in vivo* mimicking microenvironment, assisting the development of *in vitro* disease modeling^7–11^.

### Collagen microencapsulation

Our group have previously established a collagen-based microencapsulation platform which entraps living cells within a reconstituted nanofibrous collagen meshwork, providing a biocompatible and physiologically relevant microenvironment for cell attachment, proliferation, migration and differentiation^12^. In a previous report, our group also demonstrated that chondrocytes from OA patients exhibited phenotypic changes when co-culture with mesenchymal stem cells (MSCs)^13^. This suggests that chondrocytes inside the collagen meshwork are able to sense and respond to extrinsic factors. It also suggests the potential for collagen microspheres to act as an *in vitro* model to study OA.

In this study, we aim to evaluate the phenotypes of primary human osteoarthritic chondrocytes (hOACs) when they are microencapsulated in 3D collagen microspheres. As reference, we compare the chondrocytes phenotypes with those under the traditional 2D monolayer culture and 3D pellet culture model. Specifically, we aim to study the structural change, expression of major chondrogenic, osteoarthritic and hypertrophic markers, and cellular deposition of extracellular matrix particularly glycosaminoglycan (GAG) and collagen II in these *in vitro* models so as to evaluate the ability of these *in vitro* models in restoring and retaining the OA chondrocyte phenotypes. Moreover, we would like to demonstrate the phenotypic changes of hOACs in collagen microspheres when exposed to a few external factors including serum-free medium, hypoxia and transforming growth factor beta (TGF-β). These phenotypic changes are compared with those reported by previous studies, in order to reveal the ability of 3D collagen microsphere model as an *in vitro* screening model for disease-modifying treatments.

## Results

### Morphological characterization of OACs in collagen microspheres and pellets

**Figure 1:**
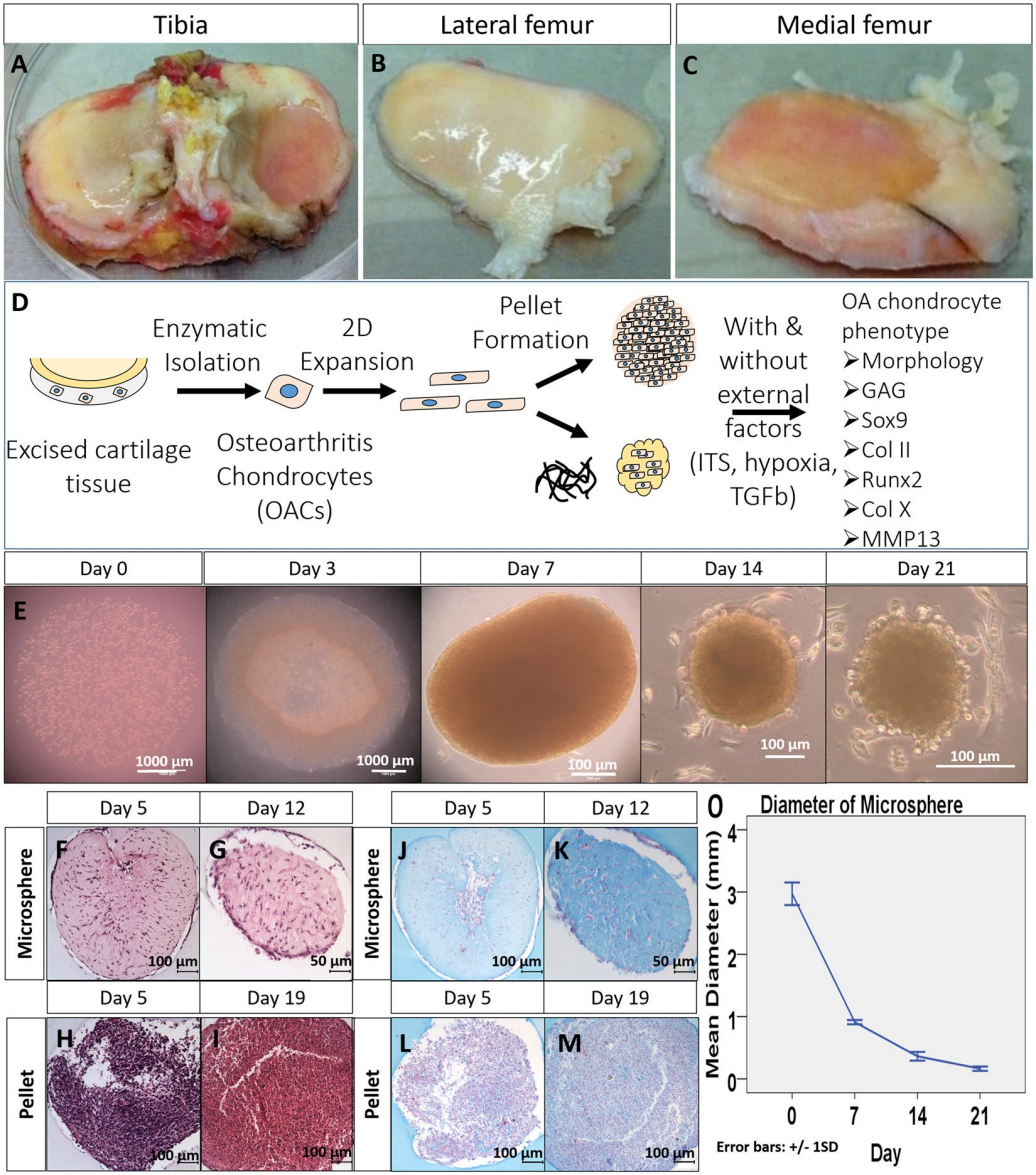
General experimental design and gross appearance, H&E and Alcian blue staining of chondrocyte encapsulated collagen microspheres, compared with pellet. Excised tissues from tibia plateau (**A**), lateral (**B**) and medial femoral condyle (**C**) were collected from total knee replacement surgery. Schematic diagram of the overall experimental design (**D**). In brief, chondrocytes in the cartilage were enzymatically isolated from the cartilage specimens and expand in monolayer culture. The cells (P3) were then cultured in 3D in order to resume its *in vivo* characteristics. Two kinds of 3D culture method, namely pellet culture and collagen microencapsulation were used for chondrocyte 3D culture and the effect in phenotypes restoration were compared. Collagen microspheres at different time points during a 3 week culture (**E**). H&E staining (**F**–**I**) and Alcian blue staining (**J**–**M**) of microsphere and pellet. Diameter of microsphere during a 3 week culture (**O**), from 5 independent experiments, each with diameter measurement of 30 microspheres at each time points.

The appearance of collagen microspheres experienced notable changes during cultures. Figure 1E show the gross appearance of OACs in the microspheres at day 0, 3, 7, 14, 21 respectively. The spherical appearance was maintained over time but with an obvious contraction, as shown by Fig. 1O. Human OA chondrocytes were embedded in collagen and GAG containing matrix (Fig. 1F–K). As shown by Fig. 1H,I, chondrocyte pellets were highly packed with little extracellular matrix (Fig. 1H,L). Unlike microsphere, enlargement of pellets (data not shown) was observed that may be related to cell proliferation or matrix deposition.

### Expression of chondrogenic markers by OACs in collagen microspheres and pellets

As shown by Fig. 1J,K, OACs in microspheres were able to deposit GAG-rich matrix as the alcian blue staining increased in microsphere of later time point. For pellets, there was no significant difference in the level of staining for GAGs between the early time point and the later time point (Fig. 1L–M).

**Figure 2:**
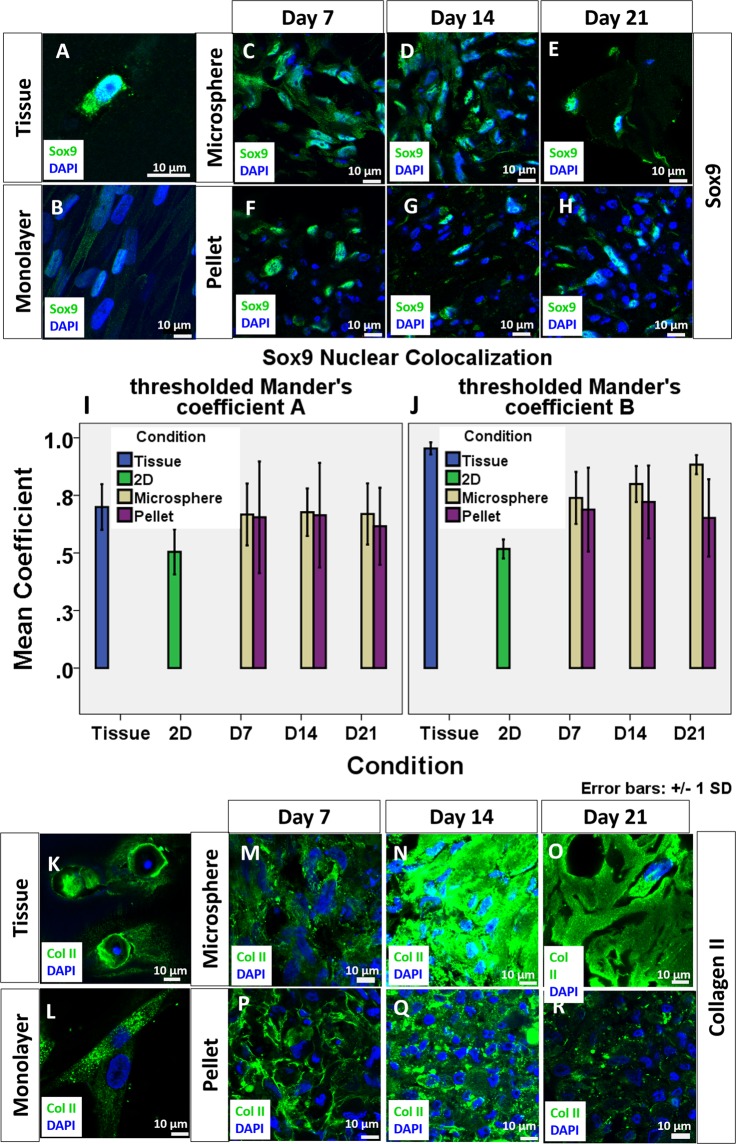
Expression of chondrogenic markers of chondrocytes in cartilage, monolayer culture, collagen microspheres and pellet at different time points. SOX9 expression in chondrocytes of OA cartilage specimen (**A**), monolayer cultured chondrocytes (**B**), chondrocytes collagen microspheres (**C**–**E**) and pellet (**F–H**). Green: SOX9; Blue: DAPI. Scale bar: 10 μm. The colocalization of Sox9 and DAPI was revealed as Manders’s Colocalization Coefficient (MCC). The coefficient is calculated as the fraction of Sox9 in compartment containing DAPI (**I**) and the fraction of DAPI in compartment containing Sox9 (**J**). Each reading is the mean from three independent experiments, each with 5 images. Collagen II expression of chondrocytes in OA cartilage specimen (**K**), monolayer culture chondrocytes (**L**), chondrocytes collagen microspheres (**M**–**O**) and pellet (**P–R**). Green: Collagen II; Blue: DAPI; Scale bar: 10 μm.

Sry-related HMG box 9 (SOX9) was universally observed in chondrocytes of native osteoarthritic cartilage specimens. As shown by Fig. 2I,J, the Manders’ Colocalization Coefficients (MCC) showed a high degree of colocalization in chondrocytes in the native cartilage tissue, suggesting strong activation of SOX9. In the monolayer culture group, low level of SOX9 activation was revealed by a low MCC with respect to both SOX9 and DAPI. When the cellular environment was resumed back to 3D (Fig. 2C–H), both microsphere and pellet led to an upregulation of SOX9 and its activation as shown by the enhanced level of SOX9 in the nucleus and hence an increased MCC value. Activated SOX9 could be observed throughout the 3 weeks culture period as shown by both the staining and the colocalization coefficient. For MCC of SOX9, two way ANOVA analysis with Bonferroni post hoc test did not find significant difference between microspheres and pellets and among different time points (p > 0.05). This implies that SOX9 level is comparable in both 3D culture methods. However, the extensiveness of expression was different between microspheres and pellets. The expression of SOX9 was extensive in most cells in collagen microspheres (Fig. 2C–E) but only around one-fifth cells (data not shown) in pellets are expressing SOX9 (Fig. 2F–H). In both cartilage tissue and monolayer culture, collagen II expression was observed. As shown by Fig. 2K,L, the collagen II expression was found to be at the circumference of the lacunae and extracellular space in the native cartilage tissue while cells in the monolayer culture express collagen II in the cytoplasm. When the cells were cultured in 3D, collagen II was consistently deposited at the extracellular space in both pellet culture and 3D microspheres.

### Expression of OA phenotypes by chondrocytes in collagen microspheres and pellets

**Figure 3:**
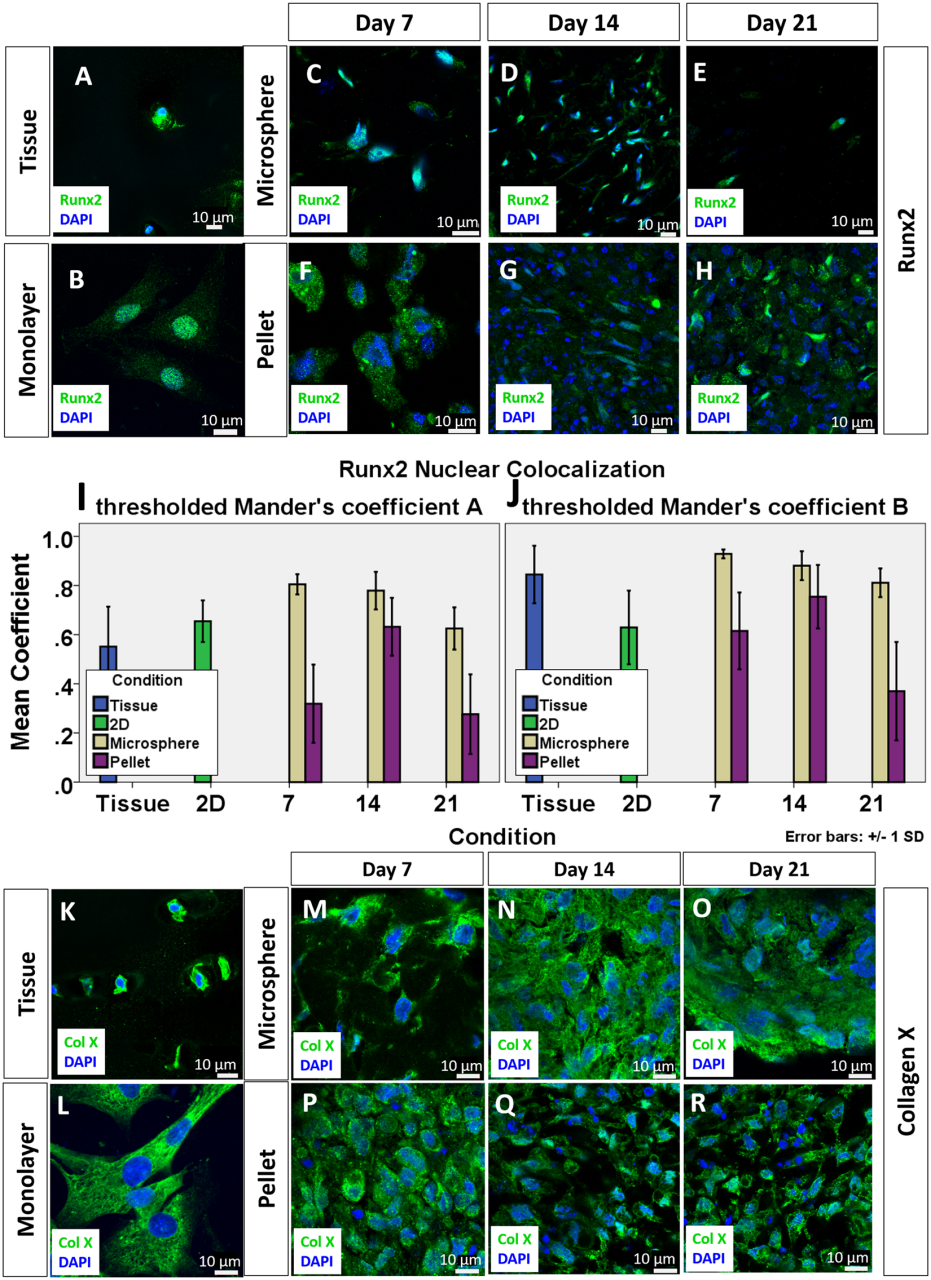
Expression of OA phenotypes of chondrocytes in cartilage, monolayer culture, collagen microspheres and pellet of different time point. RUNX2 expression in chondrocytes of OA cartilage specimen (**A**), monolayer culture chondrocytes (**B**), chondrocytes collagen microspheres (**C**–**E**) and pellet (**F–H**). Green: RUNX2; Blue: DAPI. Scale bar: 10 μm. The colocalization of Runx2 and DAPI was revealed as Manders’s Colocalization Coefficient. The coefficient is calculated as the fraction of RUNX2 in compartment containing DAPI (**I**) and the fraction of DAPI in compartment containing Runx2 (**J**). Each reading is the mean from three independent experiments, each with 5 images. Collagen X expression of chondrocytes in OA cartilage specimen (**K**), monolayer culture chondrocytes (**L**), chondrocytes collagen microspheres (**M**–**O**) and pellet (**P**–**R**). Green: Collagen X; Blue: DAPI; Scale bar: 10 μm.

As a good *in vitro* disease model, the OA phenotype should also be well retained. It is believed that the OACs resemble certain features of terminal differentiating chondrocytes or hypertrophic chondrocytes^14,15^. Therefore, the expression of three hypertrophic markers, Runt-related transcription factor 2 (RUNX2), collagen X and Matrix metallopeptidase 13 (MMP13) are used as the relevant OA phenotypic markers in the current study. Other than being a hypertrophic marker, MMP13 is prevalent in osteoarthritic joint responsible for degrading type II collagen and it is generally believed as one of the OA phenotype markers^16^. As shown by Fig. 3A, RUNX2 expression was evident in chondrocytes of the native OA cartilage, suggesting hypertrophic characteristics of the cells. Colocalization of the RUNX2 signal with the nucleus indicates RUNX2 activation in OA tissues. Figure 3B shows the sustained expression of Runx2 in monolayer culture. Majority of the RUNX2 signal was colocalized with DAPI, as depicted by the high MCC value (Fig. 3I,J). Upon 3D culture, RUNX2 was still observable in both microspheres and pellets. Pellet has a lower MCC values when compared to microspheres. Two-way ANOVA with Bonferroni post-hoc test shows a statistically significant difference between the microsphere and the pellet groups (p < 0.001) and at different time points (p < 0.001). Additionally, RUNX2 expression in pellet was mainly in the cytoplasm, as shown by Fig. 3F–H, suggesting that the RUNX2 might not be activated. As shown by Fig. 3K, another hypertrophic marker collagen X was extensively expressed in the native OA cartilage tissue, again suggesting the cellular hypertrophic feature in OA cartilage. In the monolayer culture, the expression of collagen X was extensive (Fig. 3L). Such collagen X expression was also maintained in the 3D culture groups, as shown by Fig. 3M–R. When the cells were encapsulated into 3D environment, collagen X was still evident.

### Gene expression of the chondrogenic and OA phenotype markers

**Figure 4:**
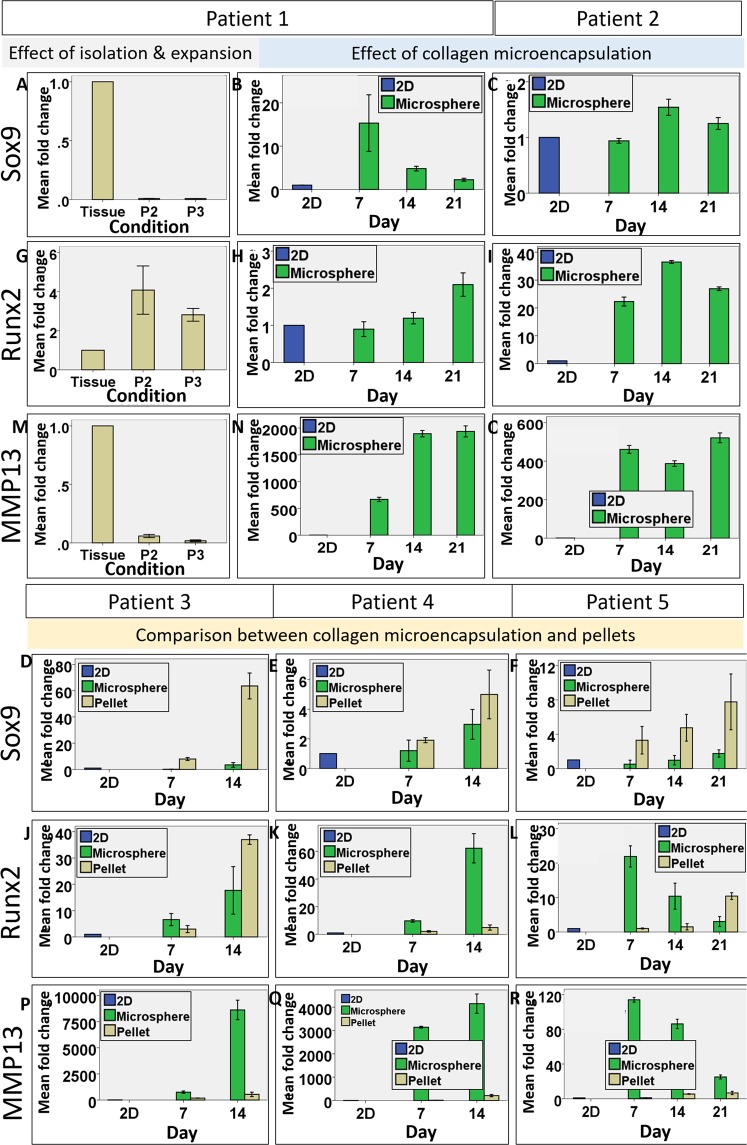
Bar charts showing the gene expression of Sox9, Runx2 and MMP13 by chondrocytes of 5 patients in collagen microspheres and pellets over time. The phenotypic changes of Patient #1 cells upon enzymatic digestion and *in vitro* expansion was revealed by significant change in the expression level of *SOX9* (**A**), *RUNX2* (**G**) and *MMP13* (**M**). The effect of collagen microencapsulation on the gene expression of *SOX9* (**B,C**), *RUNX2* (**H,I**) and *MMP13* (**N,O**) in Patients #1 and 2’s cells was evaluated over a 3 week culture period. The effect of microsphere and pellets culture in restoring *SOX9* (**D**–**F**), *RUNX2* (**J–L**) and *MMP13* (**P–R**) was compared. All error bar: +/− 1 SD. Fold changes reading are means from technical triplicates.

qPCR was conducted to reveal the cellular changes in the gene expression of *SOX9*, *RUNX2* and *MMP13* in multiple patient samples, at different time points, using monolayer culture and native cartilage tissue as references. First, the phenotypic changes of cells upon enzymatic digestion and *in vitro* expansion was revealed by significant change in the expression level of the 3 genes. Figure 4A,G,M show the change in *SOX9*, *RUNX2* and *MMP13* gene expression of Patient #1’s OACs in monolayer culture (Passage 2–3), fold changes were normalized to the level of the native tissue. Both *SOX9* and *MMP13* was tremendously downregulated after isolation and expansion. *SOX9* expression dropped to around 1% of the *in vivo* level in P2 and P3 cells, while *MMP13* dropped to around 6% in P2 cells and further down to around 2% in P3 cells. In contrary, *RUNX2* was significantly upregulated by the processes by 4-fold and 3-fold in P2 and P3 cells respectively. These results suggested that OACs could not maintain the chondrogenic phenotype at all once they are isolated and expanded.

When P3 cells from 2 patients (Patient #1 and 2) were then microencapsulated to form microspheres, the gene expression of all three markers were increased. Figure 4B,C,H,I,N,O show the fold change of gene expression in microspheres, normalized to respective 2D cells in monolayer cultures. Upregulation in all 3 genes was observed although the expression pattern varied in the 2 patients. Patient #1’s cells showed more than 10 fold increase in *SOX9*, 2 fold increase in *RUNX2* and nearly 2000 fold increase in *MMP13*. Patient #2’s cells showed 1.5 fold increase in *SOX9*, 40 fold increase in *RUNX2* and around 500 fold increase in *MMP13*. The upregulation of *SOX9* and *MMP13* in the collagen microspheres suggested partial resume of the OA phenotype. However, the level of gene expression in *SOX9* is still lower than that in the native cartilage while the levels in *RUNX2* and *MMP13* expression exceeded that of the native OA tissue.

In separate experiments using cells from Patients #3-5, the chondrocyte and OA phenotype maintenance of OACs cultured in 3D collagen microspheres and pellets were compared. Cellular gene expression in pellets was compared with that in microsphere over 2 week (Patient #3) and 3 week period (Patients #4 and 5). Figure 4D–F,J–L,P–R show the fold change of *SOX9*, *RUNX2*, *MMP13* gene expression, normalized to the level of the respective P3 cells cultured in monolayers. Generally, both 3D culture methods upregulated *SOX9*, *RUNX2* and *MMP13* comparing with that of the 2D monolayer cultures, again suggesting a better mimic of the chondrocyte and OA phenotype of cells from native OA cartilage, albeit patient-to-patient variance was noted. Depicted by Fig. 4D–F, the pellets showed a higher *SOX9* expression than the collagen microspheres in all 3 patients’ cells and 2 way ANOVA showed that the differences among different patients are significant (Patient #3: p < 0.001, patient #4: p < 0.05, patient #5: p < 0.001). And both 3D culture methods have a stronger effect in later time points, suggesting a time-dependent resume of the chondrocyte phenotype. In contrary, the collagen microspheres generally better retained the OA phenotype markers, *RUNX2* and *MMP13*. Two-way ANOVA with Bonferroni post hoc test showed that the difference in *RUNX2* between the two groups was significant in patient 3 (p < 0.001) and 5 (p < 0.001). Two-way ANOVA showed that the difference in *MMP13* in all 3 patients were also significant (p < 0.05).

In summary, OACs cultured in 3D pellet culture mimic better the *in vivo* chondrogenic phenotype as compared to the native OA cartilage tissues while OACs cultured in the 3D collagen microspheres retained better the OA phenotype.

### Effect of serum-free culture medium on hOACs in collagen microspheres

**Figure 5:**
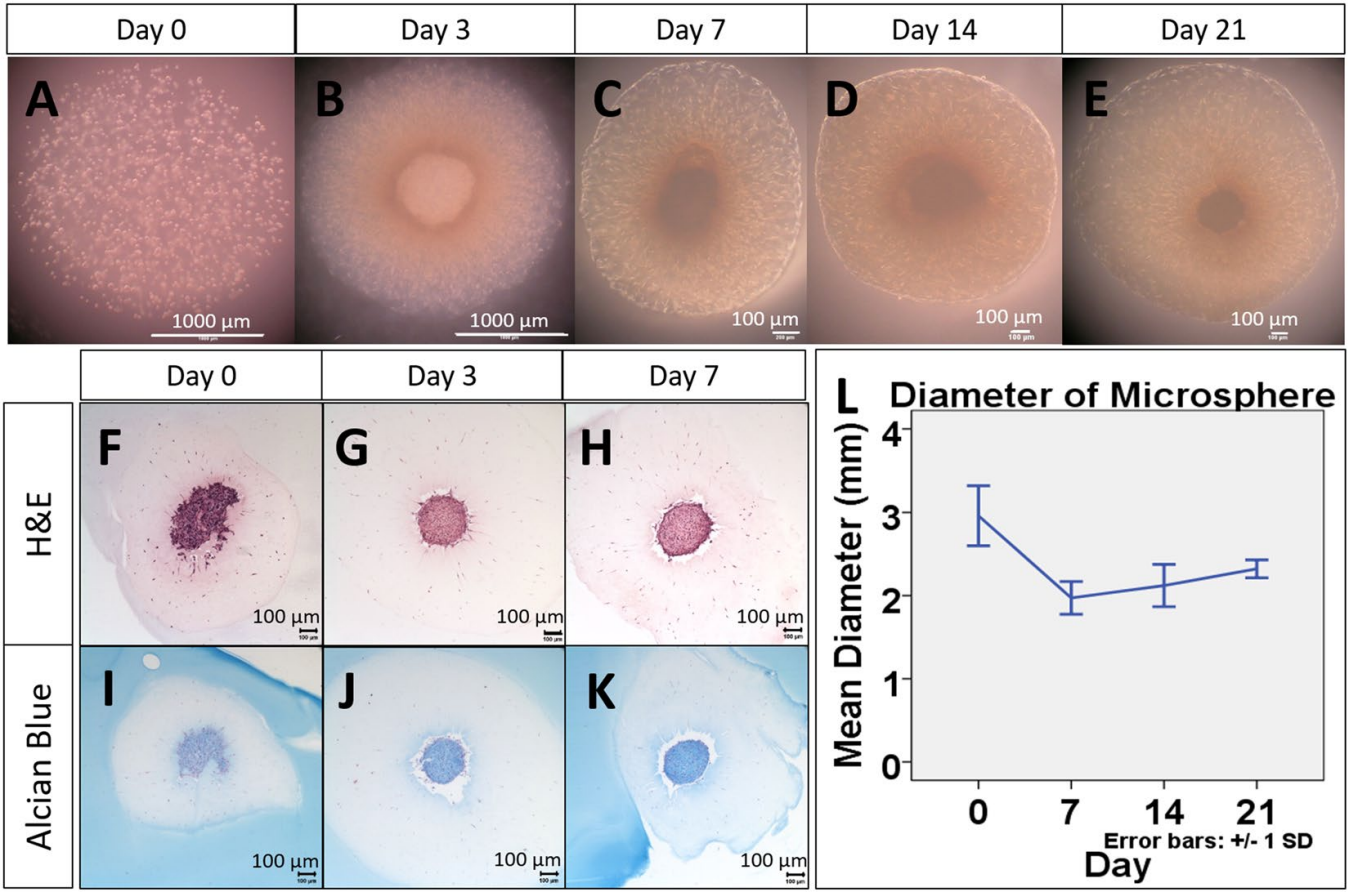
Gross appearance, H&E staining and Alcian blue staining of serum-free medium cultured chondrocyte collagen microspheres of different time points. Collagen microspheres were cultured in serum-free medium for different period of time. H&E staining (**F–H**) and Alcian blue staining (**I–K**) of collagen microspheres cultured in serum-free medium for different period of time. The change in diameter of microsphere cultured with serum-free medium (**L**), from 4 independent experiments, each with diameter measurement of 30 microspheres at each time points.

Serum has been shown in multiple studies that it is inferior in chondrocyte culture, and a serum-free medium should be used instead^6,17–21^. In order to test the responsiveness of the *in vitro* model to external factors such as culture medium, cells from 4 patient samples (Patients #2-5) were cultured in either a serum-free medium supplemented with insulin, transferrin and selenium and dexamethasone (ITS medium), or serum (FBS) supplemented medium before phenotypic characterization. Figure 5A–E show the gross appearance of OACs in 3D microspheres cultured in serum-free medium at different time points. Unlike those cultured in FBS supplemented medium, the size of the microsphere did not change significantly over time and their diameter was maintained at around 2 mm, as shown by Fig. 5L. Cells were concentrating at the center of the microspheres in the serum-free ITS group (Fig. 5F–H) but randomly distributed in the FBS group (Fig. 1F,G). Moreover, it is interesting to observe that cells did not reside at the periphery of the microsphere, where nutrients and oxygen are usually optimal. The OACs concentrating at the center of the microspheres were also able to deposit GAG-rich matrix as shown by the increasingly intensive Alcian blue staining (Fig. 5I–K).

### Chondrogenic phenotypes of hOACs when cultured with serum-free medium

The expression of SOX9 was upregulated in OACs in collagen microspheres when cultured with serum-free ITS medium as compared with that in full serum (FBS) supplemented medium (Fig. 6.1A–C. For comparison, the SOX9 staining of OACs in microspheres cultured in FBS-containing medium was shown in Fig. 6.1D–F, which duplicated Fig. 2C–E). With serum-free ITS medium, intensive whitish signal showing the co-localization of SOX9 in the nuclear region of most cells throughout the 3 weeks culture period (Fig. 6.1A–C) was observed, suggesting activation of SOX9. Two-way ANOVA with Bonferroni post hoc test show that the difference in MCC values between the ITS and the FBS groups is insignificant, suggesting that switching the culture medium into a serum-free ITS medium did not significantly affect the chondrogenic phenotype of human OACs. Collagen II was also actively deposited in the extracellular space of the collagen microspheres when serum-free ITS medium was used. As shown by Fig. 6.1I, intense signal was observed in the central region where cellular density is high, indicating active collagen II deposition in the core region. The magnified views of the central core, as depicted by Fig. 6.1J, further showed that the signal was prominent around cells.

**Figure 6:**
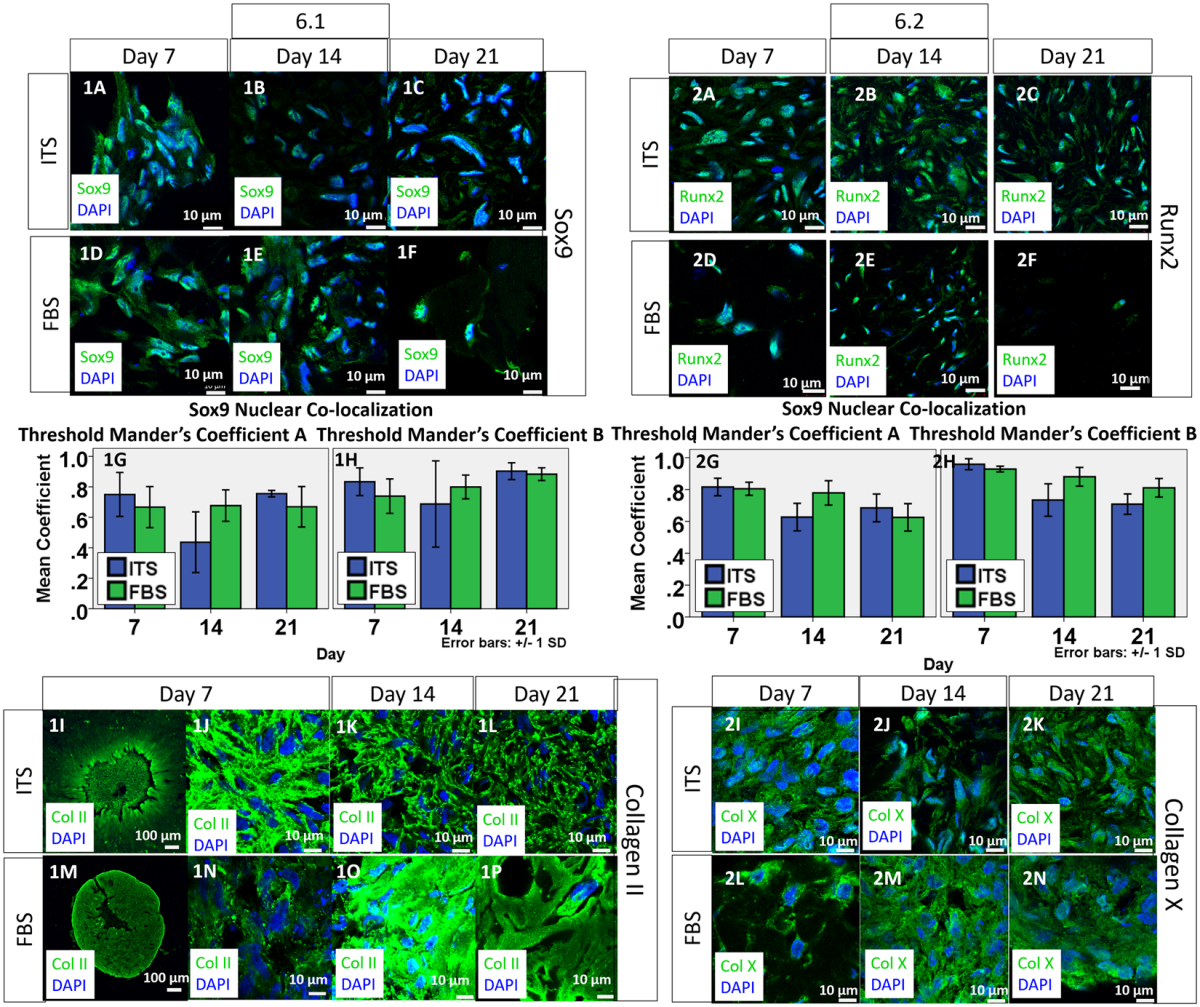
Chondrogenic and hypertrophic phenotypes of chondrocytes in collagen microspheres cultured in serum-free medium in different time point. 6.1 SOX9 expression of chondrocytes in collagen microspheres cultured with serum-free (ITS) medium (**1A–C**) and serum-containing (FBS) medium (**1D–1F**). Green: SOX9; Blue: DAPI. Scale bar: 10 μm. The colocalization of SOX9 and DAPI was revealed as Manders’s Colocalization Coefficient. The coefficient is calculated as the fraction of SOX9 in compartment containing DAPI (**1G**) and the fraction of DAPI in compartment containing SOX9 (**1H**). Each reading is the mean from three independent experiments, each with 5 images. Collagen II expression of chondrocytes in collagen microspheres cultured with ITS medium (**1I–1L**) and FBS medium (**1M–1P**). Green: Collagen II; Blue: DAPI; Scale bar: 10 μm. 6.2 RUNX2 expression in chondrocytes of collagen microspheres cultured with ITS medium (**2A–2C**) and FBS medium (**2D–2F**). Green: RUNX2; Blue: DAPI. Scale bar: 10 μm. The colocalization of RUNX2 and DAPI was revealed as Manders’s Colocalization Coefficient. The coefficient is calculated as the fraction of RUNX2 in compartment containing DAPI (**2G**) and the fraction of DAPI in compartment containing RUNX2 (**2H**). Each reading is the mean from three independent experiments, each with 5 images. Collagen X expression of chondrocytes chondrocytes collagen microspheres cultured with ITS medium (**2I–2K**) and FBS medium (**2L–2N**). Green: Collagen X; Blue: DAPI; Scale bar: 10 μm.

### OA phenotypes of hOACs when cultured with serum-free medium

Like serum cultured microsphere, both OA phenotype markers RUNX2 and collagen X were notably expressed throughout the culture period. Fig. 6.2A–F shows the immunofluorescent staining of RUNX2 in OACs in collagen microspheres cultured in ITS or FBS medium (For comparison purpose, Fig. 6.2D–F duplicated Fig. 3C–E). Two-way ANOVA with Bonferroni post hoc test indicate significant difference between ITS and FBS medium (p < 0.001) and among three different time points (all p < 0.05) on MCC value (Fig. 6.2G,H). Figure 6.2I–N show the immunofluorescent staining of collagen type X in OAC-collagen microspheres cultured in ITS or FBS medium (For comparison purpose, Fig. 6.2L–N duplicated Fig. 2M–O). Collagen X expression was also evident in the ITS group over the 3 weeks culture period, similar to that of FBS group.

### Gene expression of hOACs when cultured with serum-free medium

**Figure 7:**
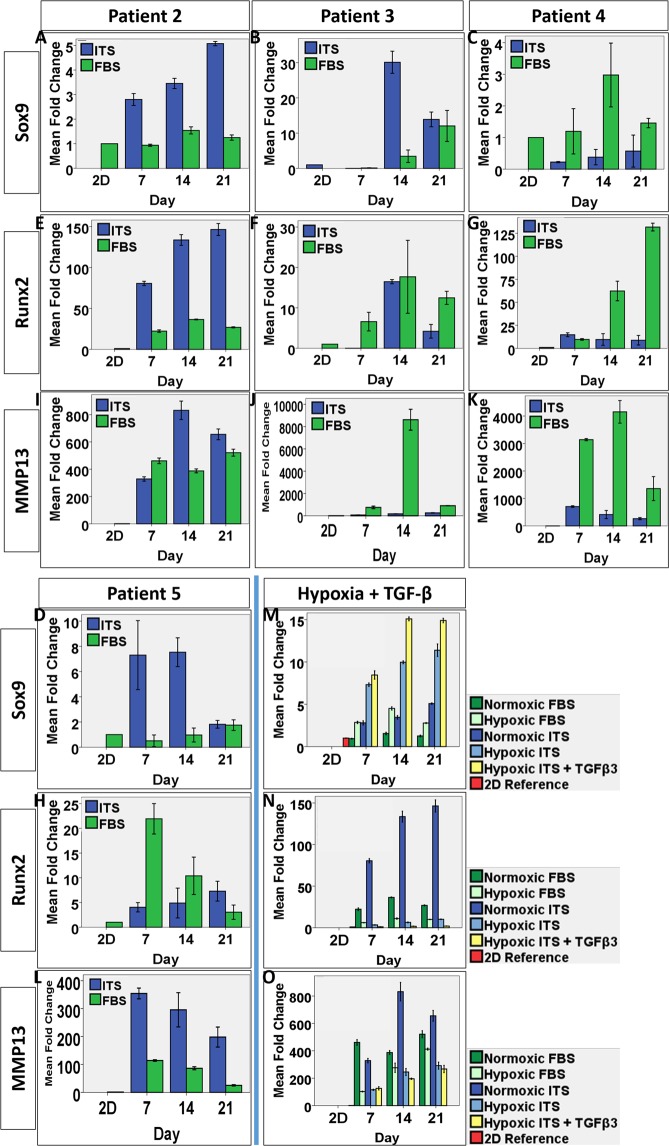
Bar charts showing the gene expression of *SOX9*, *RUNX2* and *MMP13* by collagen encapsulated chondrocytes cultured under different conditions. The effect of collagen microencapsulation with ITS or FBS culture medium on the gene expression of *SOX9* (**A–D**), *RUNX2* (**E–H**) and *MMP13* (**I–L**) in Patients #2, 3, 4 and 5’s chondrocytes was evaluated over a 3-week culture period. The effect of hypoxia and TGF-β on the gene expression of *SOX9* (**M**), *RUNX2* (**N**) and *MMP13* (**O**) in Patient # 2 chondrocytes was evaluated over a 3-week culture period. All error bar: +/− 1 SD. Fold changes reading are presented as means from technical triplicates.

The effect of ITS medium on *SOX9*, *RUNX2* and *MMP13* gene expression was quantitatively compared with that of FBS medium culture microspheres by qPCR (FBS data was duplicated partly with Fig. 4D–F,J–L,P–R for comparison). As shown by Fig. 7A–L, the change of gene expression in ITS or FBS groups was normalized to the level of respective monolayer cultured cells. Samples from 4 patients (Patients #2-5) were analyzed. Comparing with the FBS group, the serum-free ITS group further upregulate the expression of the master chondrogenic marker *SOX9* in 3 out of 4 cases (Patients #2, 3 and 5), as shown by Fig. 7A,B,D. Two-way ANOVA with Bonferroni post-hoc tests indicate significant difference between the two medium and among different time points in these 3 patients (all p < 0.001). For the OA phenotype marker *RUNX2*, the FBS group showed higher gene expression than the ITS group in 3 out of 4 cases (Patients #3-5) as shown by Fig. 7F–G. Two-way ANOVA with Bonferroni posthoc test indicate significant difference between the two medium group and among different time points in two patients (Patients #4 and 5, all p < 0.001). Notably, ITS group of patient 2 has significantly higher *RUNX2* expression than FBS group and 2 way ANOVA with Bonferroni posthoc test indicate significant changes between the two group (p < 0.001) and among different time points (p < 0.001). *MMP13* expression was dramatically upregulated in both ITS and FBS groups as compared with their 2D counterparts. With serum free ITS medium, 2 out of 4 (half) cases showed, consistently, lower expression of *MMP13* than the FBS group (Patients #3-4), two-way ANOVA with Bonferroni posthoc tests showed that the differences were statistically significant (both p < 0.001). Nevertheless, the ITS groups of other 2 patients (Patients #2 and 5) showed higher *MMP13* expression than the FBS group and two-way ANOVA with Bonferroni post-hoc tests showed that the difference was significant (both p < 0.001).

### Responses of hOACs in 3D collagen microspheres towards extrinsic factors

Additional tests were conducted using one patient’s chondrocytes (Patient #2) to evaluate the cellular responsiveness in collagen microspheres to low oxygenation level^22^ and TGF-β^23^, which are known to be chondrogenic. *SOX9* gene expression was shown to be upregulated by the induction of low oxygenation (2%) culture environment and supplementation of TGF-β (Fig. 7M). On the other hand, both *RUNX2* and *MMP13* level was downregulated by hypoxia (Fig. 7N,O). In summary, the current 3D culture model (human OA chondrocytes microencapsulated in collagen microspheres) is able to respond to external factors such as the presence of serum in the culture medium by altering the chondrogenic and the OA phenotypes at both protein and gene expression levels.

## Discussion

This work evaluated the hOACs phenotype in 3D collagen microspheres culture, and compared the phenotypic maintenance of hOACs in this model with that in the 2D monolayer culture, the 3D pellet culture and the native OA tissue. It also demonstrates the responsiveness of hOACs in 3D collagen microspheres when exposed to external factors such as serum-free medium, hypoxia and TGF-β, corroborating with previous studies^6,17–19,21–23^.

Pellet is a well-established 3D culture for chondrocytes in different *in vitro* models^24–27^ as its high cellular density could resemble mesenchymal condensation^28,29^, and are able to restore the *in vivo* phenotypes to certain extent. The simplicity of processing is another reason for its popularity. However, pellets start with cells only and it takes time for cells to deposit their own neo-ECM and becoming biomimetic. Due to limited tissue sources, scarce cellularity in cartilage and limited proliferating ability of chondrocyte *in vitro*, compressing a large number of primary cells into a single pellet is not economical and could be challenging in making patient-derived models.

In order to overcome such difficulties encountered by pellet culture, different methods are being used to culture primary chondrocytes. Microcarrier with rotating wall vessels present a promising way^30,31^. However, this kind of bioreactors approach require highly specialized devices that might not be accessible to every groups. Biomaterial scaffold 3D culture methods, on the other hand, provide a non-instrumentation dependent approach in achieving the goal^32^. Synthetic biomaterials could offer a flexible platform for chondrocytes culture with tailored-made properties for different cell types^33^. Nevertheless, this approach requires optimization of a vast range of material properties, as well as minimization of cytotoxicity and maximization of biocompatibility. In contrary, using natural polymers could offer a much simpler approach and avoid the headache of cytotoxicity and biocompatibility issues. Fibrin glue^8^, collagen^34^, chitosan^8^ have been proven to be good choices for *in vitro* cell cultures. Our study provides evidence to support the use of collagen scaffold in establishing 3D culture for hOACs.

Besides, the key features of the 3D microsphere and the 3D pellet models are compared (Table 1). In brief, the 3D collagen microsphere platform is largely comparable with the 3D pellet model but have multiple advantages such as high throughput in fabrication (thousands of microspheres can be made with ease), biomimetic cellular microenvironment (higher cell-matrix interactions) and physiologically relevant cell density (relatively lower cell density) that is comparable with native cartilage tissues. Moreover, the 3D collagen microsphere platform also better maintains the OA phenotype of the human OACs in terms of higher *RUNX2* and *MMP13* level. Additionally, the 3D collagen microsphere model enables the encapsulated OACs to respond to external factors.

**Table 1:** Comparison between collagen microsphere and pellet cultures.

	3D culture method		Pellet	Microspheres	
				FBS	ITS
Phenotypic features	Production Throughput		Low	**High**	**High**
	Cell-matrix interactions		Low	**High**	**High**
	Cell-cell interactions		**High**	Low	Low
	Cell density		**High**	Low	Low
Chondrogenic phenotype	GAG staining		Low	**High**	**High**
	Sox9	staining	High (but only a few cells)	**High (Most cells)**	**High (Most cells)**
		gene expression	**+++**	++	**+++ (3/4 patients increased)**
	Col II	staining	++ (less extracellular deposition, 3^rd^ week very low)	**+++**	**+++ (high extracellular deposition)**
OA phenotype	Runx2	staining	++ Low activation	**+++ High activation**	**+++ High activation**
		gene expression	+	**++**	+(but ¼ highly upregulated)
	Col X	staining	++	**++ (more extracellular deposition)**	**++**
	MMP13	gene expression	+	**+++**	++ (but 2/4 patients upregulated)

Additionally, 3D collagen microsphere model is a versatile platform for human OA studies. Various parameters of the collagen microencapsulation including dimension of the microsphere, collagen concentration and cell density could be manipulated to further optimize the biomimicry of the human OACs using native OA cartilage tissues as reference. Apart from the factors we tested, inflammatory cytokines and presence of mechanical loading, could be incorporated into the *in vitro* model to verify the responses of the hOACs to these well-known etiological factors. Testing the responsiveness of hOACs to these well-known extrinsic factors could provide further insights into the OA therapeutics development.

Direct comparison between the hOACs and healthy chondrocytes from human samples would certainly yield the most convincing evidence in evaluating *in vitro* 3D culture models. However, the clinical accessibility of such healthy cartilage is the hurdle to such comparative studies. As a result, exploring the possibility to collect relatively fresh samples from cadavers is warranted in future studies.

In conclusion, our study has demonstrated that hOACs resumed their *in vivo* phenotype and better recapitulated the OA phenotypes when encapsulated in the 3D collagen microsphere model, supporting the usage of the collagen microencapsulation as an *in vitro* culture platform for hOACs. Additionally, we have exemplified the application of this platform as a potential screening platform for different disease modifying agents for development of OA therapeutics.

## Methods and Materials

### Overall experiment design

As depicted by Fig. 1D, chondrocytes were enzymatically isolated from the excised cartilage specimens and cultured as monolayer for expansion. The cells at the third passage (P3) were then detached and cultured in 3D, either as pellets or in collagen microspheres. The chondrogenic and OA phenotypes of the human OA chondrocytes were characterized. The expression of chondrogenic master regulator Sox9 and cartilage specific protein collagen II was used to evaluate whether the de-differentiated cells could resume chondrogenic phenotypes while the expression of MMP13, Runx2 and collagen X was used to define the OA phenotype.

### Isolation and culture of human osteoarthritic chondrocytes (hOACs)

Sample processing and chondrocyte isolation were performed according to previously established protocol^13^ and was approved by Institutional Review Board Patient consent, sample harvesting and preparation were compliant with an institutional board (IRB) approved protocol (Hospital Authority Hong Kong West Cluster) with informed consent collected from each patient. In brief, human osteochondral specimens were collected from 5 patients (60–80 years old, all female) suffering from end-stage osteoarthritis and undergoing total knee replacement. Figure 1A–C show the typical gross appearance of the specimens collected. Part of the cartilage was excised and fixed by 4% PFA at 4 °C overnight, acting as the control for the subsequent immunofluorescent staining. Isolation of chondrocytes was carried out using the remaining cartilage specimens. Cartilage samples were minced into 1 mm thick and digested with 0.2% pronase (Sigma-Aldrich) for 1.5 hour, followed by 0.1% collagenase (Sigma-Aldrich) for 7 hours at 37 °C. Cell suspension was filtered through a 70 µm cell strainer (BD Sciences, Franklin Lakers, NJ, USA). After centrifugation and cell counting, chondrocytes were re-suspended and seeded on culture dishes at a density of 5e5 cells per 10 ml of culture medium in a 100 mm culture dish. The culture medium composed of Dulbecco’s modified Eagle’s medium (DMEM) containing 10% FBS, 0.4 µM proline, 50 µg/ml ascorbic acid, 10 mM HEPES, 0.1 mM non-essential amino acids, 2 mM L-glutamine and 1% Antibiotic-Antimycotic (Thermo Fisher). Culture medium was changed twice a week.

### 3D collagen microencapsulation of hOACs

Chondrocytes were microencapsulated in collagen as previously reported^12^. In brief, chondrocytes were cultured to sub-confluence and were then detached by treating with 0.25% trypsin-EDTA (1X) (Gibco) for 5 minutes. Rat tail type I collagen (BD) was neutralized by 0.1 N NaOH and diluted into final concentration of 1 mg/ml. Chondrocytes were suspended in neutralized collagen solution to make up the cell-matrix mixtures with final cell density of 4e5 cells/ml. Liquid droplets (50 μl) of the cell-matrix mixtures were dispensed onto a non-adhesive surface, which is UV-irradiated parafilm in a 100-mm Petri dish, and then incubated at 37 °C with 5% CO_2_ for 45 minutes to induce gelation. Gelated collagen microspheres were gently flushed with culture medium into a Petri dish and maintained as suspensions. Culture medium was changed twice a week.

### 3D pellet culture of hOACs

As a control group, chondrocytes were cultured in 3D pellets for subsequent evaluation. Each pellet was formed by centrifugation of 4e5 chondrocytes in 500 µl of culture medium at 300 rcf for 5 minutes in centrifuge tubes^6^. After 72 hours, the pellets were gently aspirated from the tubes into 24-well plate (Iwaki), with one pellet in a well maintained in 2 ml of culture medium. Each well was wrapped by UV-irradiated parafilm to prevent cells from adhering to the bottom. Culture medium was changed twice a week.

### Preparation of serum-free medium

To test the responsiveness of the collagen microspheres to external factors, one condition used was to culture the hOSC-collagen microspheres in a serum-free ITS medium. The phenotypic maintenance was compared with that cultured with full medium with 10% FBS. The serum-free culture medium was composed of 1% ITS-A (Thermo Fisher), 0.4 µM proline, 50 µg/ml ascorbic acid, 10 mM HEPES, 0.1 mM non-essential amino acids, 2 mM L-glutamine and 1% Antibiotic-Antimycotic (Thermo Fisher).

### Morphology and histology

Phase contrast images of collagen microspheres at different time points during a 3-week culture: Immediate after encapsulation (Day 0), 3 days, 7 days, 14 days and 21 days were taken. The diameter of microspheres during culture was measured weekly. Chondrocytes microspheres and pellets were fixed in 4% PFA (Santa Cruz) at room temperature in dark for 30 minutes followed by dehydration, paraffin embedding and sectioning into 10 µm thick paraffin sections. Routine H&E staining and Alcian blue staining of sections of microspheres harvested at day 5, day12 and pellets at day 5, day 19 were conducted.

### Immunofluorescence staining

Monolayer cell culture, Chondrocytes microspheres and pellets were fixed in 4% PFA (Santa Cruz) at room temperature in dark for 30 minutes. Cartilage tissues were fixed in 4% PFA (Santa Cruz) at room temperature in dark overnight. Except monolayer culture, other samples were cryo-embedded and cut into 20 µm thick frozen sections. The sections were first incubated overnight at 4 °C with primary antibodies against Sox9 (ab76997), collagen II (ab34712), Runx2 (ab76956) and Collagen X (ab49945) respectively, followed by incubation with an Alexa Fluor 488-conjugated secondary antibodies (Abcam) in dark for 30 minutes. Fluorogel with Dapi (EMS, USA) is used to mount cover glass on sections and counterstain the nucleus. Images were taken using an inverted confocal microscopy (LSM710, Carl Zeiss, Germany).

### Image analysis

The colocalization of transcription factor (Sox9 or Runx2) and nucleus (DAPI) in the region of interest was calculated by the built-in function of Imaris (Bitplane) as Manders overlap coefficient. To exemplify, the colocalization of two channel R (red) and G (green) is calculated as respective functional overlap, which is the fraction of R pixels (containing G signal) and the fraction of G pixels (containing R signal) as shown by the equation.

$${M}_{1}=\frac{{\sum }_{i}\,{R}_{i,colonal}}{{\sum }_{i}\,{R}_{i}}\,{M}_{2}2=\frac{{\sum }_{i}\,{G}_{1,colonal}}{{\sum }_{Gi}\,Gi}$$

### RNA extraction and real-time qPCR

Chondrocytes microspheres and pellets were firstly washed thoroughly and the total RNA was extracted using Tri-reagent (MRC, USA) according to manufacturer’s protocol. RNA was quantified using a NanoDrop^TM^2000 spectrophotometer. cDNA was formed using Takara PrimeScript^TM^ RT reagent Kit with gDNA Eraser (Takara) according to manufacturer’s protocol. mRNA level was determined by ABI StepOnePlus^TM^ Real-Time PCR system using Taqman probes including Sox9 (assay ID: Hs00165814_m1), Runx2 (assay ID: Hs00231692_m1) and MMP13 (assay ID: Hs00233992_m1). Passage 3 cells were used as 2D control if not otherwise specificed.

### Statistical analysis

Quantitative results including microsphere diameter, gene expression level were presented as mean ± standard deviation if not otherwise specified. The normality assumption was verified before parametric testing was used. Two-way ANOVA with appropriate post hoc tests were used to reveal statistically significant differences among different groups and subjects. The significant level was set at 0.05 and SPSS 19.0 (IBM) was used to execute the statistical analysis.
